# Celiac disease in Iranian irritable bowel syndrome patients; a systematic review and meta-analysis 

**Published:** 2019

**Authors:** Milad Azami, Gholamreza Badfar, Ghobad Abangah, Leily Mahmoudi

**Affiliations:** 1 *Medical Student, Student Research Committee, School of Medicine, Ilam University of Medical Sciences, Ilam, Iran *; 2 *Assistant Professor, Department of Pediatrics, Behbahan School of Medicine, Ahvaz jundishapour university of Medical science, Behbahan, Iran *; 3 *Department of Gastroenterology and Hepatology, Faculty of Medicine, Ilam University of Medical Sciences, Ilam, Iran *; 4 *Faculty of Medicine, Dezful University of Medical Sciences, Dezful, Iran *

**Keywords:** Celiac disease, Irritable bowel syndrome, Meta-Analysis, Iran

## Abstract

**Aim:**

The present study was conducted to evaluate the prevalence, clinical symptoms and pathological findings of celiac disease (CD) in irritable bowel syndrome (IBS) patients in Iran.

**Background:**

Several studies show high prevalence of CD in IBS patients, but the results are contradictory.

**Methods:**

The present study was conducted based on MOOSE protocol and results were reported according to PRISMA guideline. The search was done using international online databases (Scopus, PubMed, Science Direct, Cochrane Library, Embase, and Web of Science), national databases and Google Scholar search engine.

**Results:**

The pooled prevalence of CD in 2,367 Iranian IBS patients was estimated to be 6.13% (95%CI: 4.11-9.05). The prevalence of CD in men and women with IBS was 4.28% (95% CI: 2.45-7.37) and 7.19% (95% CI: 4.51-11.28), respectively. The serological prevalence of anti tTG-IgA (11 studies with 2901 IBS patients) and AGA-IgG (4 studies with 936 IBS patients) was estimated to be 5.35% (95%CI: 3.60-7.89) and 6.35% (95%CI: 2.05-18.03), respectively. The clinical symptoms of CD among IBS patients included predominant diarrhea (47.87% [95%CI: 22.46-74.43]), predominant constipation (17.34% [95%CI: 9.17-30.35]), and alternative diarrhea and constipation (27.84% [95%CI: 11.57-53.23]). According to pathological findings based on marsh classification, the prevalence of CD at stages 1, 2 and 3 were 30.89% (95%CI: 13.25-56.68), 36.56% (95%CI: 21.74-54.45) and 52.87% (95%CI: 14.48-88.13), respectively.

**Conclusion:**

In the present meta-analysis, we observed a high prevalence for CD among Iranian IBS patients, which is higher than global estimates. Examination of all IBS patients in terms of CD seems to be necessary, but cost-effectiveness should be considered*.*

## Introduction

 Celiac disease (CD) or gluten-sensitive enteropathy is an enteropathy associated with the immune system. Gluten in wheat, barley and oats may damage the small bowel and ultimately make it difficult to absorb nutrients (1). The prevalence of CD in the general population of Iran was reported to be 0.73% in a systematic review and meta-analysis (2). This disease is more common during childhood, adolescence and even adulthood. It should be noted that about 20% of patients who are diagnosed with this disease are above 60 years of age (3). CD has a various manifestations, including abdominal pain, chronic diarrhea, vomiting, constipation, pallor, foul-smelling stool, or fatty stool and weight loss, while almost all of them are secondary to malnutrition (4). However, it should be noted that the disease has various natural histories, and thus, the onset of symptoms varies from the first year of life to the eighth (5). Anti-gliadin IgG antibody (AGA-IgG), anti-endomysial antibody (EMA-Ab) may be observed in these patients. Anti-tissue transglutaminase (anti tTG) antibodies were more specifically used for diagnosis in this disease (with a sensitivity of 94% and a specificity of 95%) (6). Sampling of the second part of the duodenum is carried out if serological tests are positive. The biopsy of small bowel in people with CD has a specific shape. In normal condition, the small bowel contains small finger-like projections of tissue called villi that increase the surface area of the bowel. However, in patients with CD, this condition is lost and the bowel surface becomes flattened. Modified marsh classification of histologic findings are used for definitive diagnosis of CD (7-8). 

Irritable bowel syndrome (IBS) is a gastrointestinal disorder characterized by changes in bowel movements and abdominal pain without detectable structural abnormalities (9). IBS is one of the most common gastrointestinal disorders in the world and in addition to therapeutic costs, absence from school and work place causes a lot of financial losses. It also has a negative effect on the quality of life among patients (10). The prevalence of IBS in Iranian population is reported to be 1.1 to 25% (11). There are no specific tests for the diagnosis of IBS, so the definitions of the disease are based on clinical manifestations and are defined based on Rome II, III and IV (12-13). 

Considering the similarity between the symptoms of the two diseases, they co-exist in each other’s differential diagnosis, especially in cases where symptoms of CD are present at older ages (14). Several studies show high prevalence of CD in IBS patients, but the results are contradictory (15-26). Therefore, a structured review of all the documents and their combination may lead to a more comprehensive picture of the dimensions of the disease in Iranian society. One of the main goals of meta-analysis, which is a combination of different studies, is to reduce the difference between parameters due to the increasing number of studies involved in the analysis process, and one of the other important goals of meta-analysis is to address the issues of non-consistent results and their causes (27-29). Therefore, the present study was conducted to investigate the prevalence, clinical symptoms and pathological findings of CD among Iranian IBS patients using systematic review and meta-analysis.

## Methods

**Study protocol**

The present study was conducted based on Meta-analysis of Observational Studies in Epidemiology (MOOSE) guideline and results were reported according to Preferred Reporting Items for systematic reviews and meta-analyses (PRISMA) guideline (29).

**Search strategy**

Seven international online databases (Scopus, PubMed, Science Direct, Cochrane, Embase, Web of Science, and Google Scholar) were systematically searched. In addition to the articles published in English, we searched Persian articles in Iranian databases, including: Barakat Knowledge Network System (http://health.barakatkns.com), Iranian Research Institute for Information Science and Technology (IranDoc) (https://irandoc.ac.ir), Regional Information Center for Science and Technology (RICST) (http://en.ricest.ac.ir/), Magiran (http://www.magiran.com/), Iranian National Library (http://www.nlai.ir/), Scientific Information Database (SID) (http://www.sid.ir/), with similar strategy and relevant Persian keywords. The following keywords were used alone or in combination with other keywords: "Celiac Disease"(MeSH), "Prevalence" (MeSH), "Epidemiology" (MeSH), "Prevalence" (MeSH), "Irritable Bowel Syndrome" (MeSH), "Iran" (MeSH). The studies were published without time limit until April 2018. All references related to the subject were reviewed. As an example, PubMed search strategy was as follows: (“Celiac Disease” [Title/Abstract] OR “Irritable Bowel Syndrome”[Title/Abstract] OR “IBS”[Title/Abstract] OR (“Prevalence”[Title/Abstract] OR “Epidemiology” [Title/Abstract] OR “Frequency” [Title/Abstract]) AND (“Iran”[Title/Abstract]).

**Inclusion and exclusion criteria**

Inclusion criteria according to PICO (Population or Patient, Intervention, Comparison and Outcome; related to Evidence-Based Medicine): (1) **P**opulation: The epidemiologic studies on the Iranian IBS patients in English and Persian; (2) **I**ntervention: Serological tests such as anti tTG-IgA, AGA-IgG and histopathology to confirm CD and ROME criteria to confirm IBS ; (3) **C**omparison: The ones that show the prevalence of CD in both genders; (4) **O**utcome: The primary outcome was the pooled prevalence, clinical symptoms and pathological findings of CD among IBS patients and secondary outcome was pooled prevalence of CD among IBS patients based on IBS diagnosis criteria, year and quality of studies. 

Exclusion criteria: (1) non-random sample size, (2) lack of relevance to the subject, (3) study groups other than IBS patients, (4) lack of celiac examination among IBS patient, (5) non-Iranian studies, (6) case reports, review articles, congresses, letter to the editor without data and theses.

**Figure 1:**
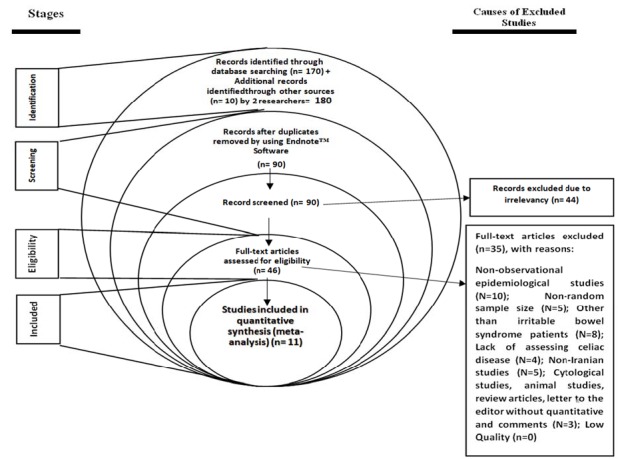
PRISMA flow diagram

**Diagnosis of IBS and CD**

IBS diagnosis was performed based on ROME II, III or IV criteria and CD diagnosis was performed based on serologic examination and pathologic confirmation (6, 9). 

**Study selection**

After omitting duplicate studies, the title and abstract of the remaining articles were reviewed by two of the authors. Studies that clearly had the exclusion criteria (did not use IBS samples or did not mention CD) were excluded. Then all the authors read the remaining studies, and the studies that had the inclusion criteria were selected, and the disagreement between the authors was resolved with the help of the third author. 

**Data extraction**

Two authors (M. A, L.M) independently extracted the data related to the characteristics of each study (such as design, demographic characteristics, diagnostic procedures, prevalence estimates, etc.) from the full text of eligible articles. Another author (Gh. B) reviewed the extracted data and resolved any disagreement. We tried to contact the authors of studies with lost or incomplete data, and this way we fulfilled the deficiencies.

The following data were extracted from eligible studies: 1. The name of the first author, 2. year of publication, year and place of study, 3. sample size, 4. reported CD prevalence, 5. CD prevalence based on serology, 6. CD prevalence based on pathology, 7. prevalence of cases 4, 5, and 6 based on gender, 8. criteria for definitive diagnosis of CD, 9. criteria for definitive diagnosis of IBS, 10. Prevalence of CD based on prevailing manifestations of IBS, 11. Prevalence of CD based on modified marsh Classification of histologic findings, 12. Recruitment setting, 13. Recruitment method, and 14. Mean age (Mean ± SD). 

**Qualitative assessment**

The Modified Scale of Newcastle Ottawa (NOS) was used to assess the quality of studies (30). The NOS uses three categories of Selection (Maximum 5 stars), Comparability (Maximum 2 stars), and Outcome (Maximum 3 stars) to evaluate bias in cross-sectional studies. The studies were divided into three categories based on the scores: studies with high risk (scores ranging from 1 to 4 stars), moderate risk (scores ranging from 5 to 7 stars), and low risk (scores ranging from 8 to 10 stars). Studies with low and moderate risk entered the meta-analysis process. 

**Statistical analysis**

Meta-analysis was done using the Comprehensive Meta-Analysis Software (CMA) version 2. The prevalence was demonstrated based on the event rate. 95% confidence interval (CI_S_) was calculated in CMA software using sample size (n) and standard error (SE). To calculate female-to-male odds ratio (OR), we used the event rate of CD in total female/female subjects to event rate of CD in total male/male subjects, finally the OR index was estimated to examine female-to-male ratio. Cochran's Q test and I^2^ index were used to determine the heterogeneity between the studies. There are three categories for index I^2^: I^2^ index below 25% is low heterogeneity, between 25-49% is moderate heterogeneity, 50-74% is substantial heterogeneity and above 75% is considered high heterogeneity (31, 32). The random effects model was used for all meta-analyses. To find the cause of heterogeneity between the studies, subgroup analysis was done based on IBS diagnosis criteria, year and quality of studies. Moreover, mixed-effects meta-regression was used to evaluate the effect of continuous variables such as the time of study on CD prevalence to deal with heterogeneity. Sensitivity analysis was done by excluding one study at a time to calculate the predictive values. Furthermore, cumulative analysis was preformed based on date of publication. Finally, publication bias was evaluated using funnel plot and Begg and Egger's test. The significance level was considered to be P < 0.05. 

## Results

**Search results and characteristics of the studies**

Overall, 180 studies were identified in systematic review. The reviewers screened the titles and abstracts, and 90 duplicate studies and 44 non-related studies were identified. Of the remaining 46 studies, 11 studies entered the quantitative meta-analysis process (Figure 1). Four studies in the center, 1 studies in the west, 1 studies in the south, 2 studies in the north, and 3 study was conducted in the east of Iran. The mean age of patients with IBS was estimated to be 33.14 years (95% CI: 31.33-34.95). Other characteristics of the studies are shown in Table 1. 

**Celiac disease**

**Figure 2:**
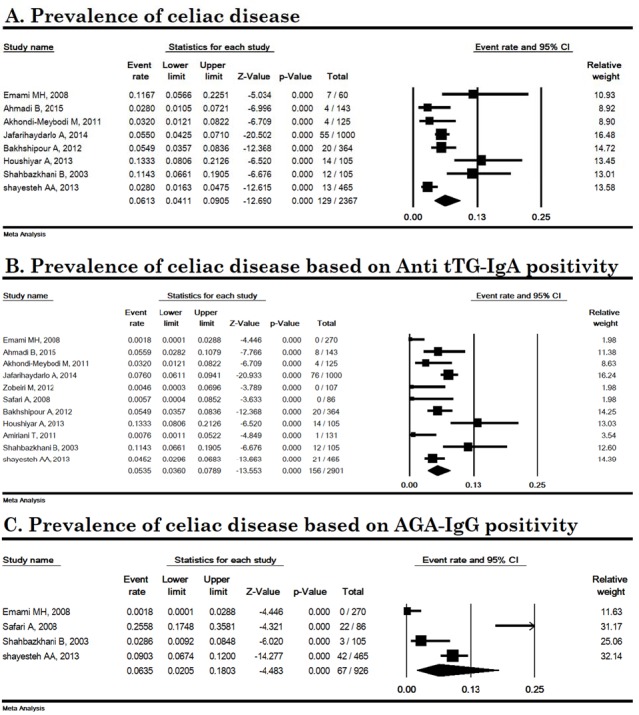
Prevalence of celiac disease in Iranian irritable bowel syndrome patients. AGA-IgG; Anti-gliadin IgG antibody, Anti tTG; Anti-tissue transglutaminase IgA antibody

In an analysis of 8 studies with a sample size of 2367, pooled prevalence of CD in Iranian IBS patients was 6.13% (95% CI: 4.11-9.05). Heterogeneity was high (I^2^ = 77.20%, P < 0.001). The lowest and highest incidences were related to the studies of Ahmadi B. *et al.* in Kerman (2.8%) and Houshiyar *et al*. in Ardabil (13.3%) (Figure 2).

**Subgroup analysis of CD prevalence**

Subgroup analysis of CD prevalence based on IBS criteria, year of study and quality of study in Table 2 showed that the difference in subgroup analysis for IBS (p = 0.008) and year of study (p = 0.001) was significant, but was not significant for the quality of study (p = 0.786) (Table 2).

**Table 1:** Summary of characteristics in studies into a meta-analysis

Quality	Sample size			Criteria for IBS	Mean Age	Place	Region	Year	First author, Published Year	Ref.
	Female	Male	All							
High			60	Rome II		Isfahan	Center	2004-5	Emami MH, 2008	
High	166	104	270	Rome II	35.3±11.8	Isfahan	Center	2004-5	Emami MH, 2008	
High	86	57	143	Rome III	34.57 ±1.24	Kerman	East	2013	Ahmadi B, 2015	
Moderate	51	74	125	Rome III	29.85±9.22	Yazd	Center	2010	Akhondi-Meybodi M, 2011	
High	503	497	1000	Rome III	29.02±11.58	Ilam	West	2008-12	Jafarihaydarlo A, 2014	18, 19
High	92	15	107	Rome II	32.68±10.22	Tehran	Center	2011	Zobeiri M, 2012	
Moderate	48	38	86	Rome III		Mashhad	East		Safari A, 2008	
High	221	143	364	Rome III	37.4±12.4	Zahedan	East	2008–10	Bakhshipour A, 2012	
Moderate	58	47	105	Rome III	31.4±10.14	Ardabil	North	2009-12010	Houshiyar A, 2013	
High			131	Rome II	31.82±10.95	Gorgan,	North	2006-8	Amiriani T, 2011	
High	73	32	105	Rome II	37.88±11.74	Tehran	Center	1999-2000	Shahbazkhani B, 2003	
High	225	240	465	Rome III	31.8±9.6	Ahvaz	South	2007-9	shayesteh AA, 2014	

**Table 2:** Subgroup analysis of celiac disease in Iranian irritable bowel syndrome patient

Pooled prevalence (%)	95%CI	Heterogeneity		Sample (N)		Studies (N)	Variable		
		P-Value	I^2^	Event	Total subjects				
11.52	7.47-17.35	0.963	0	19	165	2	Rome II	IBS detection criteria	Celiac disease
5.02	3.23-7.70	0.001	76.04	110	2202	6	Rome III		
Test for subgroup differences: Q=7.14, df(Q)= 1, P= 0.008									
7.18	2.61-18.23	<0.001	87.83	32	630	3	1999- 2007	Year of studies	
5.73	3.67-8.86	0.007	97.26	97	1737	5	2008-2012		
Test for subgroup differences: Q= 17.11, df(Q)= 3, P= 0.001									
5.73	3.80-8.55	0.002	73.77	111	2137	6	High	Quality of studies	
7.02	1.65-25.34	0.008	85.59	18	230	2	Moderate		
Test for subgroup differences: Q= 0.07, df(Q)= 1, P= 0.786									
4.28	2.45-7.37	0.031	56.81	42	1090	7	Male	Sex	
7.19	4.51-11.28	0.001	73.17	82	1217	7	Female		
The odds ratio of females to males: 1.69 (95% CI: 0.86-3.32, P=0.127), Heterogeneity: I^2^:51.38, P= 0.055									
1.19	0.12-11.04	< 0.001	84.17	13	613	4	Rome II	IBS detection criteria	Serology (anti tTG-IgA)
6.10	4.30-8.60	0.005	67.45	143	2288	7	Rome III		
Test for subgroup differences: Q= 1.95, df(Q)= 1, P= 0.162									
2.61	0.87-7.52	<0.001	80.24	34	1057	5	1999- 2007	Year of studies	
6.47	4.34-9.55	0.011	66.23	122	1844	6	2008-2012		
Test for subgroup differences: Q= 2.45, df(Q)=1, P= 0.117									
5.80	3.92-8.51	0.003	69.94	137	2454	8	High	Quality	
2.94	0.67-12.01	0.001	82.39	19	447	3	Moderate		
Test for subgroup differences: Q= 0.79, df(Q)= 1, P= 0.373									
4.14	2.02-8.31	0.015	59.94	24	712	8	Male	Sex	
4.16	1.79-9.39	< 0.001	81.43	38	972	8	Female		
The odds ratio of females to males : 1.17 (95% CI: 0.44-3.07, P= 0.746), Heterogeneity: I^2^:57.53, P= 0.051									

N; Number, CI; Confidence interval, Q; Q test for heterogeneity, df; degrees of freedom, and I^2^; I square.

**CD prevalence based on gender**

In an analysis of 7 studies, the prevalence of CD in 1090 men and 1217 women with IBS was 4.28% (95% CI: 2.45-7.37) and 7.19% (95% CI: 4.51-11.28), respectively. The OR for women and men with CD was: OR = 1.69 (95% CI: 0.86-3.32, P = 0.127) (Table 2).

**CD prevalence based on serology tests**

The serological pooled prevalence of anti tTG-IgA (11 studies with a sample size of 2901 IBS patients) and AGA-IgG (4 studies with a sample size of 926 IBS patients) were 5.35% (95% CI: 3.60-7.89) and 6.35% (95% CI: 2.05-18.03), respectively (Figure 2). The heterogeneity was high for anti tTG-IgA (I^2^ = 73.29%, P < 0.001) and AGA-IgG (I^2^ = 91.10%, P < 0.001) among CD patients. 

**Subgroup analysis of CD prevalence based on serology test**

Subgroup analysis of CD prevalence based on serology test** (**anti tTG-IgA**) **showed that the difference in subgroup analysis for IBS (p = 0.162) and year of study (p = 0.117) and quality of study was not significant (Table 2).

**Serological prevalence of CD based on gender**

The serological prevalence of anti tTG-IgA for CD was 4.14% (95% CI: 2.02-8.31) in men with IBS and 4.16% (95% CI: 1.79-9.39) in women with IBS. The female-to-male OR for CD was 1.17 (95% CI: 0.44-3.07, P = 0.746) (Table 2). 

**Sensitivity and cumulative analysis**

**Figure 3:**
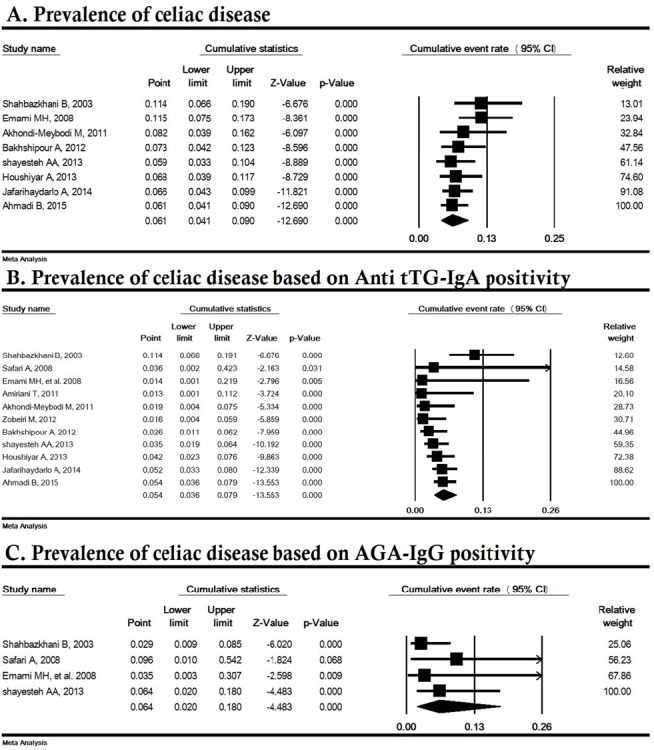
Sensivity analysis of prevalence of celiac disease in Iranian irritable bowel syndrome patients. AGA-IgG; Anti-gliadin IgG antibody, Anti tTG; Anti-tissue transglutaminase IgA antibody

**Figure 4:**
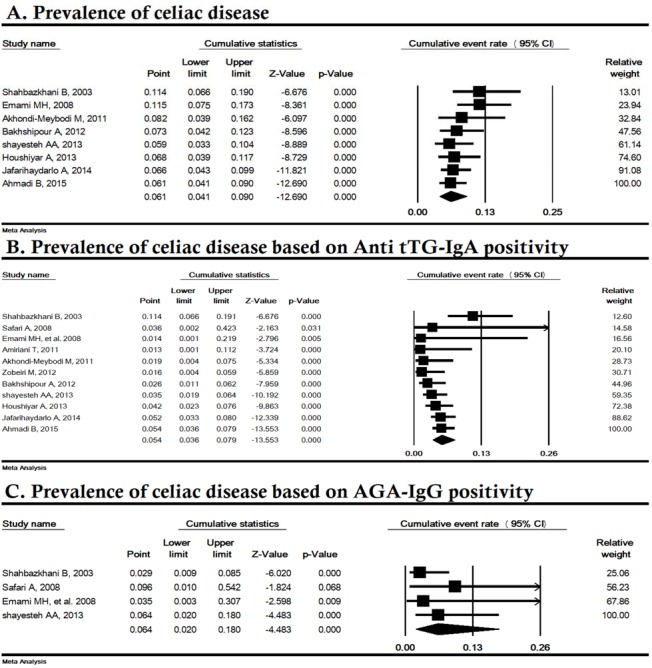
Cumulative analysis of prevalence of celiac disease in Iranian irritable bowel syndrome patients (sorted by year of publication). AGA-IgG; Anti-gliadin IgG antibody, Anti tTG; Anti-tissue transglutaminase IgA antibody

Sensitivity analysis by removing one study at a time indicated that the overall result for the prevalence and serology of CD was strong (Figure 3). The cumulative analysis based on the year of publication of the articles is shown in Figure 4; the lowest total CD prevalence and the anti tTG serology for CD were in years 2013 (5.9%) and 2011 (1.3%), respectively. 

**Figure 5:**
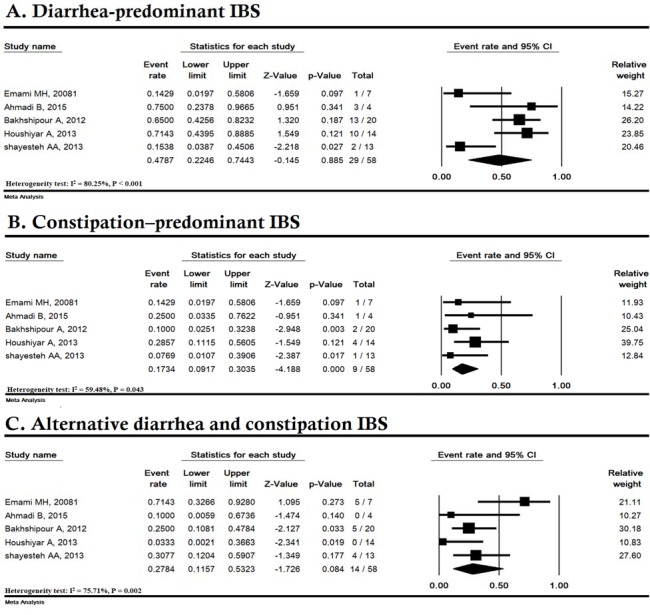
The prevalence of clinical symptoms of celiac disease among Iranian irritable bowel syndrome (IBS) patients

**Figure 6:**
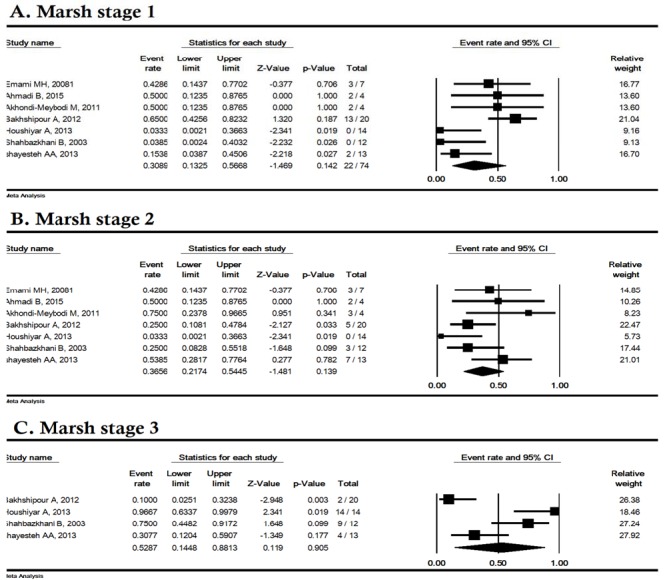
The prevalence of celiac disease according to pathological findings (based on marsh calcification) among Iranian irritable bowel syndrome patients

**Figure 7:**
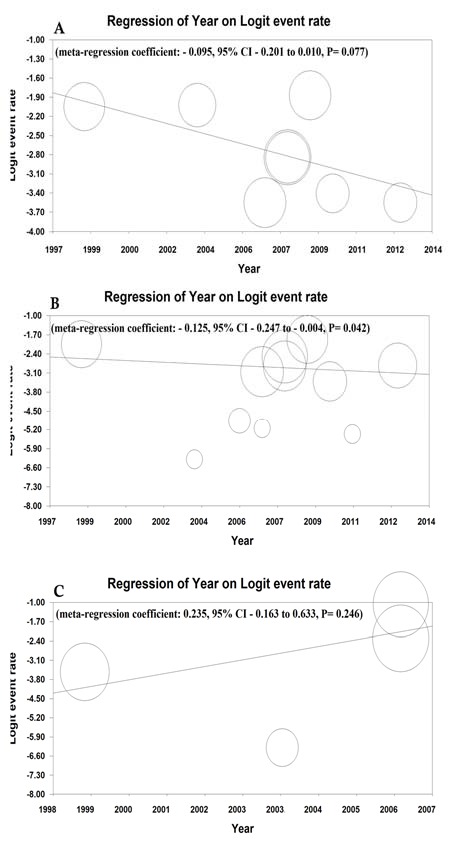
The meta-regression. Overall prevalence of celiac disease (A), serology anti tTG-IgA (B) and AGA-IgG (C) according to year of study. AGA-IgG: Anti-gliadin IgG antibody; Anti tTG: Anti-tissue transglutaminase IgA antibody

**Figure 8:**
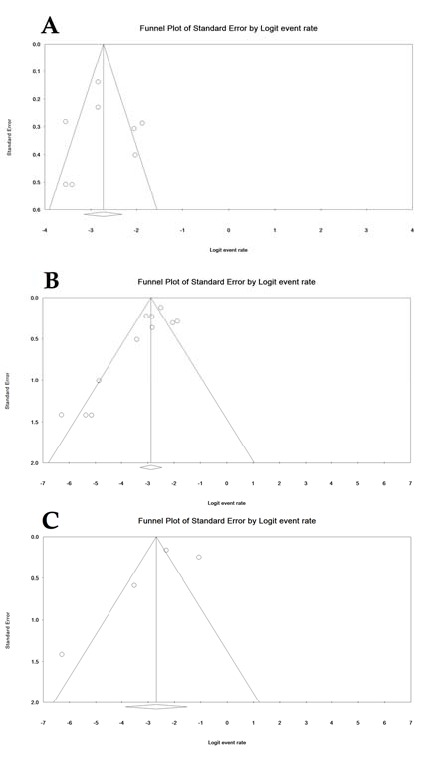
Publication bias. The overall prevalence of celiac disease (A), serology of anti Ttg-IgA (B) and AGA-IgG (C). AGA-IgG: anti-gliadin IgG antibody; Anti tTG: anti-tissue transglutaminase IgA antibody

**Clinical symptoms**

The common symptoms of CD in IBS patients were diarrhea-predominant (IBS-D) (47.87% [95% CI: 22.46-74.43]), constipation predominant (IBS-C) (17.34% [95% CI: 9.17-30.35]) and alternative diarrhea and constipation (IBS-M) (27.84% [95% CI: 11.57-53.23]) (Figure 5).

**The prevalence of pathological findings CD**

Based on marsh classification, the prevalence of CD stages 1, 2 and 3 was estimated to be 30.89% (95% CI: 13.25-56.68), 36.56% (95% CI: 21.74-54.45) and 52.87% (95% CI: 14.48-88.13), respectively (Figure 6).

**Meta-regression**

Meta-regression of CD prevalence and anti-tTG-IgA CD serology based on the year of studies had decreasing trend and the significance level was estimated to be 0.07 and 0.04, but meta-regression of AGA-IgG CD serology had decreasing trend (P = 0.24) (Figure 7). 

**Publication bias**

In the reviewed studies, publication bias was not significant for the overall CD prevalence (Egger = 0.90 and Begg = 0.90), for anti tTG-IgA CD (Egger = 0.06 and Begg = 0.11), and for AGA-IgG CD (Egger = 0.53 and Begg = 0.73) (Figure 8). 

## Discussion

 This study represents of the epidemiological aspects of CD in patients with IBS symptoms in Iran in a large scale. This study was performed to evaluate the prevalence of CD based on antibodies (tTGA and AGA-IgG), and the small bowel biopsy was performed. The serologic prevalence of tTGA and AGA-IgG CD were 5.35% and 6.35%, respectively. The CD prevalence in the pathology confirmation was 6.13% in the present study. Therefore, there was no significant difference in the prevalence of abnormal serologic tests and biopsy-proven CD. In the analysis of the causes of heterogeneity of the prevalence of CD, we can mention IBS diagnostic criteria (prevalence of CD among IBS patients according to Rome I and Rome II was estimated to be 11.5% and 5%, respectively) and the year of study (prevalence of CD among IBS patients during 1997-2007 was 2.6% and during 2008-2012 was 6.4%). The quality of studies (P = 0.78) and gender (P = 0.74) were not among the causes of heterogeneity. 

In a study among the general population of Iran, the serologic prevalence of CD (tTGA) and pathological confirmation of CD (small bowel biopsy) were 0.83% and 0.79%, respectively (2) which is much lower than the estimates of the present study. A systematic review and meta-analysis (including 14 non-Iranian studies and one Iranian study) reported IgA-class AGA, tTG antibodies (± EMA) and biopsy confirmation to be 4%, 1.6%, and 4.1%, respectively (33), which is consistent with the pathological confirmation of CD in the present study. In another meth-analysis in 2017 that reviewed 36 studies, OR for IgA AGA, and tTG antibodies (± EMA) and positive biopsy-proven CDs in IBS patients compared to control group were 3.21, 2.75, and 4.48, respectively (34). The prevalence of IBS and CD varies according to race and geographical location (7, 70-72), probably due to differences in diet, genetics and culture. 

Considering false-positive test results, it is possible that the prevalence of positive serological tests has been inflated. However, the attributes of these tests for CD diagnosis are about 95% (73), and we estimated the biopsy-proven CD rate of above 6% among those who show IBS symptoms, which was not significantly different from serologic results. Therefore, it can be concluded that the prevalence of this disease is not rare in Iran, and is higher than other countries. 

Some studies state that serologic CD tests are not routinely required in IBS patients. CD is treatable and requires gluten-free diet (39). There are wide variations in CD prevalence between different settings with different patient characteristics and various serological tests. Given these controversies, it is the time to decide on the consideration of CD serological tests in suspected IBS patients, and the cost-effective aspects should be taken into account. 

According to several studies, tests for CD in patients with the symptoms of IBS would be cost-effective (40-42). Spiegel *et al*. (41) claimed that histological examination for CD had an acceptable cost when the prevalence of CD was more than 1%, and it became the dominant strategy, and it was cheaper than empiric symptom-based therapy for presumed IBS, when the prevalence of CD reached 8%. According to another research, there was only 1% increase in lifetime costs of managing IBS with tTG tests for CD when the prevalence of CD was 3%, while the cost per quality-adjusted life-year fell to $4,900 when the prevalence of CD in IBS was assumed to be 5% (40), which is close to the estimates of our meta-analysis. Mohseninejad *et al*. (42) reported that tests for CD in patients with non-constipated IBS was almost cost-effective at a prevalence of 4.7%, which is again close to the point estimated in our study. 

Therefore, according to the up-to-date synthesis of data in the present meta-analysis, it is likely that tests for CD remains acceptable in terms of cost, although the prevalence of CD falls slightly short of making serological tests the dominant strategy.

International guidelines suggest that tTG antibody tests (±EMA testing) should be preferred over AGAs (43-46) for the diagnosis of CD due to higher sensitivity and specificity of these tests, and one needs to consider the opportunistic screening of individuals with IBS using these serological tests for CD (44-46). If we look at the data from studies that used EMA or tTG in this meta-analysis, the overall results support the screening for CD in patients subject to secondary or tertiary care. However, our results do not support the advantage of screening people at the population level, or within primary care.

The prevalence of IBS is estimated to be 10% to 20% worldwide (47-49). Patients with IBS report decreased quality of life and there are economic and social costs associated with it (48, 49). It is recommended that doctors consider positive diagnosis of IBS based on clinical features, and they currently use Rome IV criteria (47). Rome IV criteria classify IBS based on the clinical symptoms of IBS-D, IBS-C, IBS-M, or unspecified (IBS-U). In the present study, the prevalence of clinical symptoms in IBS patients with CD in more than 75% of patients was in the form of IBS-D and IBS-M. Therefore, patients with chronic diarrhea should be examined in terms of CD as one of the most important differential diagnoses. 

For future research, it is recommended that more studies be conducted in Iran to evaluate the acceptable cost of screening for CD in Iranian IBS patients.

 The limitation of this study is: 1) Lack of “AND” and “OR” operators support for a combined search in national databases; 2) Failure to investigate the prevalence of CD in IBS patients based on regions and etc due to the limited number of studies; and 3) Failure to investigate the acceptable cost of screening for CD in Iranian IBS patients due to the limited number of studies.

This meta-analysis provides an overview of CD epidemiology in IBS patients for Iranian physicians and policymakers. In the present meta-analysis, we observed the high prevalence of CD in Iranian IBS patients, which was higher than global estimates. Examination of all IBS patients in terms of CD seem to be necessary, but cost-effectiveness should be considered in screening all Iranian IBS patients in terms of CD.
